# Systemic levels of the endothelium-derived soluble adhesion molecules endocan and E-selectin in patients with suspected deep vein thrombosis

**DOI:** 10.1186/2193-1801-3-571

**Published:** 2014-09-30

**Authors:** Knut Anders Mosevoll, Roald Lindås, Øystein Wendelbo, Øystein Bruserud, Håkon Reikvam

**Affiliations:** Institute of Clinical Science, Section of Hematology, University of Bergen, Bergen, Norway; Section for Hematology, Department of Medicine, Haukeland University Hospital, N-5021 Bergen, Norway; Section for Infectious Diseases, Department of Medicine, Haukeland University Hospital, Bergen, Norway

**Keywords:** Venous thromboembolism, Deep venous thrombosis, Cytokines, Adhesion molecules, Matrix metalloproteases

## Abstract

The initial evaluation of patients with suspected deep vein thrombosis includes the use of biomarkers reflecting activation of the coagulation system. However, the thromboembolic process and neighboring inflammatory responses also affect endothelial cells, and endothelial cell markers may therefore be altered by the disease. In the present population-based single-center study, we investigated the plasma levels of the endothelium-specific biomarkers soluble E-selectin and endocan in a consecutive and unselected group of 120 patients admitted to hospital for suspected deep vein thrombosis. Blood samples were collected when patients arrived at the hospital. DVT patients showed evidence for an acute phase reaction with increased serum C-reactive protein levels, but this was similar to many other patients admitted with suspected but not verified thrombosis. Plasma endocan and E-selectin levels did not differ between patients with thrombosis, healthy controls and the patients without verified thrombosis (i.e. patients with other causes of their symptoms, including various inflammatory and non-inflammatory conditions). However, the combined use of endothelial biomarkers, C-reactive protein and D-dimer could be used to identify patient subsets with different frequencies of venous thrombosis. Thus, analysis of plasma biomarker profiles including endothelial cell markers may be helpful in the initial evaluation of patients with deep vein thrombosis.

## Introduction

The development of venous thromboembolism (VTE), i.e. deep vein thrombosis (DVT) and pulmonary embolism (PE), represents a complex interaction between inflammatory pathways as well as the coagulation system, and it involves a wide range of cells including various leukocytes, platelets and endothelial cells (Fox & Kahn 2005; Hou et al. 2012; Coleman & Wakefield 2012). The clinical presentation of DVT varies, and range from typical local signs including classical signs of inflammation to a disease with very few or no noticeable symptoms (Fox & Kahn 2005; Hou et al. 2012). The clinical handling of patients with suspected DVT can be challenging, and several molecular as well as cellular biomarkers have been suggested for the initial diagnostic handling of these patients (Hou et al. 2012; Coleman & Wakefield 2012). The biomarkers most extensively studied are molecular markers suggesting activation of the coagulation system (i.e. platelet activation, activation of the coagulation factor system) or various cellular or molecular markers indicating an inflammatory process (Hou et al. 2012; Coleman & Wakefield 2012). However, endothelial cells are also affected by and contribute to the development of DVT, and in the present study we compared systemic levels of endothelial cell markers in patients with diagnosed and suspected DVT. We then investigated the plasma levels of endocan and E-selectin as both these molecules are regarded as endothelial cell-specific. A possible future strategy for the evaluation of patients with suspected venous thrombosis might be to evaluate biomarker profiles instead of single markers.

The development of a diagnostic biomarker panel is a multistep process, as illustrated by the biomarker panel developed for diagnostic and prognostic use in acute graft versus host disease after allogeneic stem cell transplantation (Paczesny et al. 2009; Harris et al. 2012; Ferrara et al. 2011; Levine et al. 2012). The first step was to compare the levels of a large number of biomarkers between contrasting groups; the authors then compared the plasma levels of a large number of biomarkers (120 proteins) for two relatively small groups (21 patients each) with severe GVHD and without GVHD (Paczesny et al. 2009). The authors identified biomarkers showing significant differences between these two groups (35 biomarkers). In the second step they selected 23 biomarkers for further evaluation based on defined criteria, including the potential biological relevance to disease development. Thirdly, 8 selected biomarkers were tested in a large cohort of 424 unselected patients, and a panel consisting of 4 biomarkers was then found to be optimal for diagnostic and prognostic evaluation. Finally, in the last part of these studies two organ-specific biomarkers were combined with the 4 previous biomarkers to improve the diagnostic and prognostic accuracy of the panel (Harris et al. 2012; Ferrara et al. 2011; Levine et al. 2012). A similar initial strategy was also used to design a prognostic biomarker panel for patients with suspected severe sepsis, and these authors used a panel of 9 mediators in their final analysis (Shapiro et al. 2009). Several other studies also suggest a multibiomarker approach in sepsis prognostics (Skibsted & Shapiro 2014; Wong et al. 2014; Wong et al. 2012). The possible use of biomarker panels in the diagnosis of venous thromboembolic disease should probably follow a similar strategy, and the present study represents a first step in a similar process where we investigate the possible use of biologically relevant biomarkers (i.e. endothelial cell markers) in clinically relevant contrasting groups, i.e. patients with and without verified deep vein thrombosis in a group of unselected patients admitted to hospital with suspected thrombosis.

## Materials and methods

### Study population

The study was approved by the local Ethics Committee (REK Vest Norway, nr. 27.03) and patients were included after written informed consent. We included a total of 120 consecutive and thereby unselected patients with suspected DVT referred by primary health care physicians to the Emergency Department at Haukeland University Hospital. The hospital is a primary care center for all patients with suspected DVT within a defined geographic area; our study should therefore be regarded as a population-based cohort study. Our study did not include in-hospital patients who developed DVT during their hospital stay for other disorders.

All patients followed a standardized evaluation algorithm; patients with positive D-dimer (≥0.5 mg/L) and/or high Well’s score (≥2) were referred to compression ultrasound and ultimately to contrast venography if the ultrasound examination was inconclusive or a leg vein thrombosis was suspected. Control samples were obtained from 19 healthy individuals; this group included 19 adults with no disease symptoms during the last week, no present disease and none of them had taken any drugs during the last week. Due to technical reasons only plasma samples were available for 117 of the patients. Endocan levels were then investigated for all 117 patients, whereas due to technical reasons E-selectin levels were investigated for overlapping subsets of consecutive/unselected patients by ELISA and/or Luminex technology (explained in detail below in the chapter Cytokine analyses).

None of the patients without thrombosis were readmitted to hospital during the following 4 weeks with increasing or persisting symptoms requiring a new evaluation due to suspected DVT.

### Preparation and preservation of plasma samples

Peripheral venous blood samples were collected into Vacutainer 9NC tubes (Becton-Dickenson, San Jose, CA) with citric acid as anticoagulant. Samples were thereafter transferred to plastic tubes without additives and centrifuged twice at 200 g for 15 minutes at room temperature. Centrifugation was performed within 120 minutes from sampling. The plasma supernatants were finally transferred to cryotubes, frozen immediately and stored at −80°C until analyzed.

### Cytokine analyses

Soluble E-selectin (R&D Systems; Abingdon, UK; the last 98 consecutive patients) and endocan levels (Lunginnov, Pasteur Institute, Lille, France; 117 patients examined) were determined by ELISA analyses strictly according to the manufacturers’ instructions. These results were used in the clustering analyses.

E-selectin levels were in addition analyzed by Luminex analyses (R&D Systems) for the first 89 consecutive patients and for 20 healthy controls. These results were used for the comparison between healthy controls and between subsets of patients.

All results are presented as the mean of duplicates.

### Statistical and bioinformatical analyses

Statistical analyses were performed using Graph Pad Prism 5 (Graph Pad Software, Inc., San Diego, CA, USA). Pearson’s correlation test for bivariate samples was used for correlation analyses, and the Mann-Whitney’s rank sum test to compare different groups; p-values <0.05 were regarded as statistically significant. Unless otherwise stated in the text, the p-values were not corrected for the number of comparisons. SPSS version 20.0 (SPSS Inc., Chicago, Illinois, USA) was used for logistic regression analysis. Bioinformatical analyses were also performed using the J-Express 2012 analysis suite (MolMine AS) (Stavrum et al. 2008).

## Results

### DVT was diagnosed in a minority of patients admitted to hospital with suspected thrombosis

The large majority of the patients were referred to the hospital due to recent development of pain localized to the leg, pain when palpating the leg and unilateral edema of the lower extremity. The initial diagnostic evaluation of the 120 patients included clinical examination, plasma D-dimer and ultrasound/Doppler examination of the affected extremity; venography was only performed if the initial examination was inconclusive and a deep leg vein thrombosis was suspected. The D-dimer level was normal in 32 of the 120 patients. Twenty-three patients were not examined by ultrasound or venography; all of them having a normal D-dimer test and Wells score less than 2. Venography was performed in 14 patients and was positive for 2 of them. Other conditions than DVT were diagnosed by the clinical physician at the Emergency Department based on an overall evaluation of the patient. Infections were predominantly skin infections. The most important clinical data of the patients are summarized in Table 1. Median age of our patients was 68 years (range 19–101 years) with 69% females.

**Table 1:** **The final diagnoses of patients admitted to hospital with suspected deep vein thrombosis**

Diagnosis	Number of patients (n = 120)	Temperature (°C)	BP (systolic)	Heart rate	WBC (×10^9^/L)	HB (g/dL)	TPK (×10^9^/L)	Wells score
**Deep vein thrombosis**	28	36.9 (36.2-38.8)	146 (95–192)	81 (54–114)	7.8 (3.8-18.3)	14.1 (9.8-17.8)	220 (115–401)	3 (1–6)
Involving the iliac vein	10							
Involving femoral vein	13							
Only involving the leg and popliteal vein	5							
**Superficial thrombophlebitis**	6	37 (36.5-37.5)	146 (129–204)	74 (63–97)	9.3 (6.8-13.8)	14.5 (12.7-15)	257 (76–312)	1.5 (1–3)
**Sterile inflammation** including trauma/muscular bleeding, ruptured Baker’s cyst, arthritis, malignant diseases without thrombosis	33	36.9 (35.9-38.1)	150 (91–202)	81 (50–110)	8.1 (4.7-13.3)	13.9 (10.1-16.3)	292 (209–507)	1 (−2 - 4)
**Infections**	12	37.2 (36–38.5)	137 (100–169)	86 (100–169)	8.7 (5.8-14.1)	13.4 (9.7-15)	301 (200–409)	1 (−1 - 2)
**Venous stasis**	8	36.6 (36–37.5)	143 (99–183)	73 (53–104)	6.9 (4.2-12.4)	14 (10–15.9)	280 (104–528)	1.5 (0–3)
**Other conditions without inflammation** including musculoskeletal pain and neurological disorders	33	36.9 (36–38)	139 (104–175)	73 (46–96)	7.2 (2.1-13.5)	14 (8.6-16.6)	267 (179–398)	1 (−2 - 3)

Clinical data for each diagnosis are presented as median and range.

Only 28 patients were diagnosed with thrombosis; 10 patients with high thromboses involving the iliac vein, 13 patients with femoral vein and lower thrombosis, whereas a minority of 5 patients was diagnosed with leg vein thrombosis. Three of the 28 patients with DVT were also diagnosed having PE.

### Plasma endocan and E-selectin levels do not differ between patients with and without DVT

**Figure 1:**
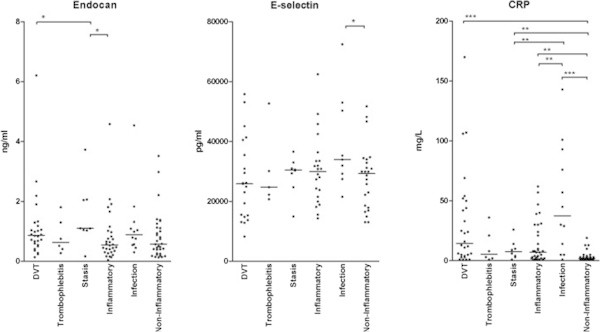
**Plasma endocan (left) and E-selectin (middle; Luminex analyses for the first 89 consecutive patients) levels and serum CRP levels (right) in patients admitted to hospital with suspected DVT; a comparison between subsets of patients with suspected thrombosis.** We investigated a group of 120 patients. The patients were classified as described in Table 1. Thrombophlebitis was defined as clinical signs of a local inflammatory reaction corresponding to a subcutaneous vein of the affected limb, infection means clinical and laboratory evidence for a local infection corresponding to the affected lower limb, and an inflammatory condition means clinical and laboratory findings consistent with a local sterile inflammation of the affected limb (e.g. a ruptured Baker’s cyst).

We compared plasma endocan and E-selectin levels for patients with DVT, patients without verified DVT and a group of 19 healthy controls. The patient results are presented in Figure 1 and the statistical analyses are summarized in Table 2; as will be described in detail below there was a large degree of overlap between patient subsets (Figure 1) and also between patients and controls (data not shown).

Endocan plasma levels were determined for all patients. When comparing the overall results the endocan levels showed a significant correlation with serum CRP (p = 0.0234) but not with D-dimer levels (p = 0.22). Plasma E-selectin levels were determined by Luminex technology for the first 89 consecutive/unselected patients, and in contrast to endocan the E-selectin levels did not show any significant correlation with CRP (p = 0.063) or D-dimer levels (p = 0.68). We also compared endocan and E-selectin (Luminex analyses, the 89 first consecutive patients) plasma levels for patients with and without deep vein thrombosis, but none of the two biomarkers differed significantly (p = 0.18 and p = 0.19, respectively). Furthermore, neither endocan nor E-selectin (Luminex analyses, the first 89 consecutive patients) showed any significant difference when comparing patients with iliac vein thromboses (n = 10) to patients with thromboses only involving the lower veins (p = 0.19 and p = 0.87 respectively). Finally, variation ranges for endocan as well as E-selectin (Luminex analyses, the first 89 consecutive patients) showed considerable overlap when comparing patients with thrombosis, patients without thrombosis and healthy individuals (Figure 1).

**Table 2:** **Comparison of serum CRP, plasma endocan and plasma E-selectin levels (luminex analyses, the 89 first consecutive patients) in patients admitted to hospital with suspected deep vein thrombosis**

	Thrombophlebitis	Sterile inflammation	Infection	Venous stasis	Other causes without inflammation
**ENDOCAN LEVELS - COMPARISON BETWEEN VARIOUS PATIENT SUBSETS (n = 117)**					
**Deep vein thrombosis (n = 28)**	0.5723	**0.0407**	0.8479	0.1058	0.1066
**Thrombophlebitis (n = 6)**		0.6558	0.3736	0.2284	0.5725
**Sterile inflammation (n = 30)**			0.0818	**0.0230**	0.8742
**Infection (n = 12)**				0.1770	0.1435
**Venous stasis (n = 8)**					0.0230
**Other causes without inflammation (n = 33)**					
**E-SELECTIN LEVELS - COMPARISON BETWEEN VARIOUS PATIENT SUBSETS (n = 89)**					
**Deep vein thrombosis (n = 21)**	0.7450	0.2485	0.0517	0.4208	0.5618
**Thrombophlebitis (n = 5)**		0.8271	0.1898	0.4351	0.8852
**Sterile inflammation (n = 22)**			0.0938	0.9813	0.5165
**Infection (n = 9)**				0.2359	**0.0499**
**Venous stasis (n = 8)**					0.5864
**Other causes without inflammation (n = 24)**					
**CRP LEVELS - COMPARISON BETWEEN VARIOUS PATIENT SUBSETS (n = 120)**					
**Deep vein thrombosis (n = 28)**	0.2132	0.0842	0.1693	0.1949	**< 0.0001**
**Thrombophlebitis (n = 6)**		0.7993	0.0608	0.8461	0.1396
**Sterile inflammation (n = 33)**			**0.0097**	1.0000	**0.0029**
**Infection (n = 12)**				**0.0277**	**< 0.0001**
**Other causes without inflammation (n = 33)**					

The Mann Whitney t-test was used for the statistical comparison and the p-value is given for each statistical comparison (significant differences are marked in bold). Thrombophlebitis was defined as clinical signs of a local inflammatory reaction corresponding to a subcutaneous vein of the affected limb, infection means clinical and laboratory evidence for a local infection corresponding to the affected the lower limb, and sterile inflammation means a condition with local sterile inflammation of the affected limb (e.g. a ruptured Baker’s cyst).

We also did the same statistical analyses for E-selectin levels determined by ELISA analyses for the last 98 consecutive patients, and the results were similar for both subsets (data not shown).

Based on the present sample size and the observed plasma levels for patients with and without verified thrombosis, there is a 20% chance (statistical power 20%; 1-p = 0.8) of not detecting a true difference and a 5% chance of detecting a false difference (α = 0.05) if the observations (i.e. Δμ) differ with endocan 0.7 pg/ml, E-selectin 8400 pg/ml, D-dimer 3.7 μg/ml and CRP 21 μg/ml.

### Plasma endocan and E-selectin levels in various patient subsets admitted to hospital with suspected deep vein thrombosis

We compared the plasma levels of soluble endocan and E-selectin for the various patient subsets admitted with suspected deep vein thrombosis, and each subset was also compared with the healthy controls. The overall results are presented in Figure 1 and the statistical comparisons are presented in detail in Table 2. There was a large overlap in plasma endocan levels between the various groups. The endocan levels for DVT patients showed a difference of borderline significance compared to patients with sterile inflammation (p = 0.0407). Patients with thromboses also differed significantly from the healthy controls, but this was also true for the other patient subsets except patients with venous stasis (data not shown).

Plasma E-selectin levels were investigated only for an unselected/consecutive subset of patients (Luminex analyses, the first 89 consecutive patients). The levels in DVT patients did not differ significantly from any other patient subset (Figure 1, Table 2). We then observed only two differences of borderline significance in plasma E-selectin levels when patients with infections were compared with (i) patients with sterile inflammation (Table 2, p = 0.0499) and (ii) with the healthy controls (data not shown). Similar results were seen when we analyzed the E-selectin levels measured by ELISA analyses for the 98 last consecutive patients (data not shown).

Finally we compared CRP levels for patient subsets and for healthy controls (Figure 1, Table 2). All patient subsets showed evidence for an acute phase reaction compared with healthy controls, and DVT patients differed significantly only from the patients without clinical evidence of inflammation.

### Combined analysis of endocan, E-selectin, D-dimer and CRP in the diagnosis of DVT

**Figure 2:**
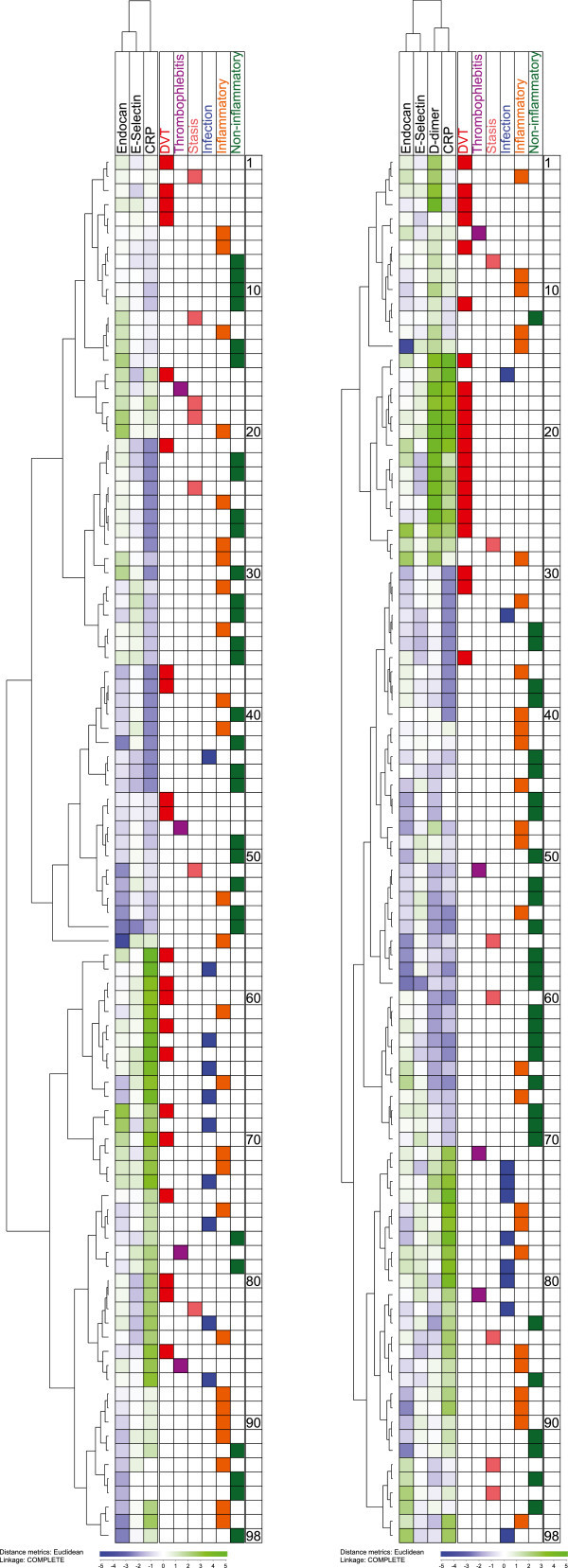
**Unsupervised hierarchical clustering of patients with suspected DVT based on serum/plasma levels of Endocan, E-selectin (ELISA analyses, the last 98 consecutive patients), CRP and D-dimer in 98 patients.** (LEFT) The clustering was based on endocan, E-selectin and CRP. (RIGHT) The clustering was based on endocan, E-selectin, CRP and in addition D-dimer.

We used unsupervised hierarchical clustering for a combined analysis of these parameters; all these analyses included the 98 consecutive patients for whom both endothelial biomarkers were analyzed by ELISA analyses. We first made an analysis only including the two endothelial cell parameters together with CRP levels (Figure 2, left part). It can be seen that the patients with DVT as well as several patients with other diagnoses were included in all except one of the main clusters; only a cluster consisting of the 11 lower patients did not include any of the DVT patients. We compared the levels of the 3 biomarkers for this subset with the other 87 patients (Table 3); the endocan plasma levels were then significantly lower than for the other 87 patients. There was a large overlap of the variation ranges for all three biomarkers between the 11 patients and the other 87 patients. Thus, the cluster is defined by the overall biomarker profile, but especially by relatively low endocan levels.

**Table 3:** **Unsupervised hierarchical clustering (see Figure** 2 **); plasma biomarker levels (median and range) for patient in clusters with low/high frequency of DVT**

**Clustering: endocan, E-selectin and CRP (Figure ** 2 **left - no DVT among patients 88–98)**				
**Mediator**	**Cluster patients 88-98**	**Cluster patients 1-87**	**p-value**	**All 98 patients**
Endocan plasma level	0.31 (0.15-0.65)	0.94 (0.04-6.2)	< 0.0001	0.82 (0.04-6.21)
E-selectin plasma level	27741 (22191–42222)	25672 (4940–60896)	ns	26286 (4940–60896)
CRP	8 (5–30)	4 (1–170)	ns	5 (1–170)
**Clustering: Endocan, E-selectin, CRP, D-dimer (Figure ** 2 **right - patients 1–29 including 18 of 21 patients with DVT)**				
**Mediator**	**Cluster patients 1-29**	**Cluster patients 30-98**	**p-value**	**All 98 patients**
Endocan plasma level	1.02 (0.04-6.21)	0.65 (0.13-4.54)	0.0102	0.82 (0.04-6.21)
E-selectin plasma level	26276 (9971–52968)	26296 (4940–60896)	ns	26286 (4940–60896)
CRP	9 (3–170)	3 (1–101)	0.0006	5 (1–170)
D-dimer	5.640 (0.90-20.00)	0.6200 (0.22-3.55)	< 0.0001	0.85 (0.22-20.00)

The Mann Whitney test was used for the statistical comparison of the two patient subsets. The concentrations are given as ng/ml for endocan, pg/ml for E-selectin and μg/ml for CRP.

We did an additional clustering analysis including endocan, E-selectin, CRP and D-dimer (Figure 2 right). The upper main cluster consisting of 29 patients included 18 of the 21 DVT patients, and the lower subcluster of this group (patients 15–29 counted from the top) included 15 patients and 12 of them had DVT. This lower 15–29 subcluster was characterized by high levels of both CRP and D-dimer. The ideal situation would be that our biomarker profile identified two major cluster where one of the clusters only included all DVT patients; with regard to this our biomarker/clustering strategy showed a specificity of 0.87 and a sensitivity of 0.86 even though the frequency of DVT patients differed significantly between the two major clusters (Figure 2 right part, p < 0.001). The corresponding values for normal versus abnormal D-dimer were sensitivity 1.00, specificity 0.38 and p = 0.003. Finally, the 29 patients in the upper main cluster differed significantly from the lower main cluster (patients 30–98) both with regard to endocan, CRP and D-dimer levels and showed increased levels of all three mediators (Table 3), but again the variation ranges showed a large extent of overlap. The identification of two main subsets is thus dependent on the overall biomarker profile, but the E-selectin levels seem to be less important.

We also used an adjusted logistic regression model combining endocan, E-selectin, D-dimer and CRP; this did not detect any additional differences for any mediator.

We conclude that the combination of two endothelial cell markers, one coagulation marker and one acute phase reactant seems to identify a patient subset with a very low risk of DVT (lower patients) and also a subset of patients with very high probability of DVT.

## Discussion

Previous studies have described that the systemic levels of several biomarkers are altered in patients with DVT compared with healthy controls. In the present study we investigated plasma levels of the endothelium-specific biomarkers endocan and E-selectin, and we describe that combined analysis of these biomarkers (either the two markers alone or together with D-dimer and CRP) could be used to identify patient subsets with different frequencies of DVT in a group of consecutive/unselected patients admitted to hospital with suspected DVT.

The design of diagnostic biomarker panels will often be a multistep process as illustrated by the diagnostic and prognostic biomarker panel for acute GVHD (Paczesny et al. 2009; Harris et al. 2012; Ferrara et al. 2011; Levine et al. 2012; Shapiro et al. 2009). In our opinion a similar strategy should be used for DVT (see the last chapter of the Introduction), and our present study suggest that only endocan but not E-selectin differ between patients with verified thrombosis and various patient subsets with symptoms due to other diseases. Our hierarchical clustering analyses further illustrates how the use of endothelium-specific biomarkers can be used in combination with other markers to identify patient subsets with a high frequency of thrombosis. The design of a diagnostic biomarker panel for patients with suspected DVT will require both larger clinical studies than our present study and should include a limited number of carefully selected biomarkers (often 4–9 markers (Paczesny et al. 2009; Harris et al. 2012; Ferrara et al. 2011; Levine et al. 2012; Shapiro et al. 2009); in our opinion endothelium-specific biomarkers should be included and endocan then seems to be most relevant.

The diagnosis of DVT is usually based on a careful clinical evaluation, D-dimer levels and visualization of the thrombosis (Goodacre et al. 2006; Tenna et al. 2012). In our present study all patients were initially evaluated with clinical examination, D-dimer and ultrasound examination; venography was performed only if DVT was still suspected but not visualized after this initial examination. By using this diagnostic approach our present study confirmed that DVT is diagnosed only in a minority of patients admitted to hospital with suspected thrombosis.

The development of venous thrombosis involves activation of the coagulation system, but there is also a local inflammatory response. The endothelial cells can be affected by both these factors. For this reason we investigated whether the endothelial cell involvement is reflected in the systemic (i.e. plasma) levels of endothelium-specific biomarkers, and whether these levels can be used in the diagnostic evaluation of DVT patients. For these reasons we examined consecutive patients admitted to hospital with suspected deep vein thrombosis, and the plasma levels of endothelial cell markers in DVT patients were compared both with other patients with suspected DVT and with healthy controls.

Endocan is an endothelium-derived proteoglycan that shows increased plasma/serum levels in different conditions, including infections and malignant diseases (Delehedde et al. 2013; Sarrazin et al. 2006; Hatfield et al. 2011). Our present study demonstrated that the plasma levels of this biomarker show a considerable overlap when comparing healthy individuals and patients with or without thrombosis. This overlap makes it difficult to use endocan alone in the diagnostic evaluation of patients with suspected deep vein thrombosis. E-selectin is also an endothelium-derived adhesion molecule that can be detected in a soluble form in serum/plasma. These levels are decreased during febrile neutropenia, whereas increased levels are detected during bacterial infections in immunocompetent patients (Bruserud et al. 1996; Bruserud et al. 1995). Our present study demonstrated that this biomarker showed only minor variation in patients with suspected venous thromboembolism.

Our present study confirmed that DVT is associated with an acute phase reaction, including increased CRP levels. However, this is a common characteristic for many patients admitted with suspected DVT, and not only for the minority who are diagnosed with a thrombosis. Many patients with suspected DVT thus have an inflammatory condition, and this is probably the reason why there is a large overlap in plasma endocan and E-selectin levels between patients with diagnosed DVT and other patients with suspected thrombosis. Plasma endocan seems to be a part of this acute phase reactions and shows a correlation with the CRP levels in patients with suspected DVT.

Soluble E-selectin levels has previously been investigated once in patients with venous thromboembolic disease (Bucek et al. 2003), and our result confirm these authors negative findings. Systemic endocan levels have only been investigated in one previous study only including patients with verified pulmonary embolism (Guzel et al. 2014). These authors described higher endocan levels in patients with massive/submassive embolism compared with nonmassive embolism. However, we could not detect any difference between patients with iliac vein thrombosis and patients with lower DVT. A possible explanation for this difference between pulmonary embolism and DVT patients could be that the increased endocan levels in patients with massive pulmonary embolism are mainly due to the embolism rather than the original thrombosis and/or the affected circulation with pulmonary hypertension/right heart failure.

We also did clustering analyses to investigate the combined use of four biomarkers in the diagnosis of DVT: two endothelial cell markers, one coagulation parameter and one acute phase reactant. By this combined approach we could identify patient subsets with very low and very high probability of DVT. These observations suggest that combined analyses may be useful in the clinical handling of patients with suspected DVT, but further analysis in future clinical studies is required.

D-dimer is the biomarker most commonly used in the initial evaluation of patients with suspected DVT, but none of the available biomarkers can be used alone to exclude the possibility of DVT (Hou et al. 2012; Coleman & Wakefield 2012). One possible strategy to improve the diagnostic strength of biomarker analyses would be to combine various biomarkers in a biomarker profile. Such a diagnostic approach would require the development of new bioinformatical tools suitable for routine clinical use, but it would also require a careful evaluation of single biomarkers with regard to their possible use as a part of a biomarker profile.

There are several limitations of our study. Firstly, our study is relatively small, and additional differences between patient subsets may have been detectable if a larger number of patients had been investigated. However, in our opinion this would have been of limited interest because of the wide variation ranges and the large overlap between patients, this makes it unlikely that these biomarkers will have a diagnostic/clinical relevance when used alone. Secondly, we only included patients from outside the hospital mainly living at home, and we do not know whether our approach will be useful for another patient population characterized by additional and more severe diseases. Thirdly, the plasma levels of endothelial biomarkers may be altered by DVT-predisposing conditions (e.g. active cancer, pregnancy, surgery), and/or the role of endothelial cells in the pathogenesis of DVT may vary between the biologically heterogeneous predisposing factors, e.g. lupus anticoagulants, genetic predisposition in the coagulation cascade, essential thrombocythemia, paroxysmal nocturnal hemoglobinuria (Van Bijnen et al. 2012; Reikvam & Tiu 2012; Uitte de Willige et al. 2008). Finally, a further evaluation of endothelial cell biomarkers in the evaluation of patients with suspected DVT would also require the development of bioinformatical tools that are suitable for use in routine clinical practice.

We conclude that DVT is not associated with increased systemic levels of endothelial cell markers (endocan, E-selectin) compared with healthy controls, but minor differences can be seen between subsets of patients with verified and suspected DVT especially for endocan. Thus, these plasma endothelial cell markers alone do not seem to be useful in the diagnostic evaluation of patients with suspected deep vein thrombosis, but they may become useful in combination with other markers.

## References

[CR1] Bruserud O, Akselen PE, Bergheim J, Nesthus I (1995). Serum concentrations of E-selectin, P-selectin, ICAM-1 and interleukin 6 in acute leukaemia patients with chemotherapy-induced leucopenia and bacterial infections. Br J Haematol.

[CR2] Bruserud O, Halstensen A, Peen E, Solberg CO (1996). Serum levels of adhesion molecules and cytokines in patients with acute leukaemia. Leuk Lymphoma.

[CR3] Bucek RA, Reiter M, Quehenberger P, Minar E, Baghestanian M (2003). The role of soluble cell adhesion molecules in patients with suspected deep vein thrombosis. Blood Coagul Fibrinolysis.

[CR4] Coleman DM, Wakefield TW (2012). Biomarkers for the diagnosis of deep vein thrombosis. Expert Opin Med Diagn.

[CR5] Delehedde M, Devenyns L, Maurage CA, Vives RR (2013). Endocan in cancers: a lesson from a circulating dermatan sulfate proteoglycan. Int J Cell Biol.

[CR6] Ferrara JL, Harris AC, Greenson JK, Braun TM, Holler E, Teshima T, Levine JE, Choi SW, Huber E, Landfried K, Akashi K, Vander Lugt M, Reddy P, Chin A, Zhang Q, Hanash S, Paczesny S (2011). Regenerating islet-derived 3-alpha is a biomarker of gastrointestinal graft-versus-host disease. Blood.

[CR7] Fox EA, Kahn SR (2005). The relationship between inflammation and venous thrombosis. A systematic review of clinical studies. Thromb Haemost.

[CR8] Goodacre S, Stevenson M, Wailoo A, Sampson F, Sutton AJ, Thomas S (2006). How should we diagnose suspected deep-vein thrombosis?. QJM: monthly journal of the Association of Physicians.

[CR9] Guzel A, Duran L, Koksal N, Torun AC, Alacam H, Ekiz BC, Murat N (2014). Evaluation of serum endothelial cell specific molecule-1 (endocan) levels as a biomarker in patients with pulmonary thromboembolism. Blood Coagul Fibrinolysis.

[CR10] Harris AC, Ferrara JL, Braun TM, Holler E, Teshima T, Levine JE, Choi SW, Landfried K, Akashi K, Vander Lugt M, Couriel DR, Reddy P, Paczesny S (2012). Plasma biomarkers of lower gastrointestinal and liver acute GVHD. Blood.

[CR11] Hatfield KJ, Lassalle P, Leiva RA, Lindas R, Wendelboe O, Bruserud O (2011). Serum levels of endothelium-derived endocan are increased in patients with untreated acute myeloid leukemia. Hematology.

[CR12] Hou H, Ge Z, Ying P, Dai J, Shi D, Xu Z, Chen D, Jiang Q (2012). Biomarkers of deep venous thrombosis. J Thromb Thrombolysis.

[CR13] Levine JE, Logan BR, Wu J, Alousi AM, Bolanos-Meade J, Ferrara JL, Ho VT, Weisdorf DJ, Paczesny S (2012). Acute graft-versus-host disease biomarkers measured during therapy can predict treatment outcomes: a Blood and Marrow Transplant Clinical Trials Network study. Blood.

[CR14] Paczesny S, Levine JE, Braun TM, Ferrara JL (2009). Plasma biomarkers in graft-versus-host disease: a new era?. Biol Blood Marrow Transplant.

[CR15] Reikvam H, Tiu RV (2012). Venous thromboembolism in patients with essential thrombocythemia and polycythemia vera. Leukemia.

[CR16] Sarrazin S, Adam E, Lyon M, Depontieu F, Motte V, Landolfi C, Lortat-Jacob H, Bechard D, Lassalle P, Delehedde M (2006). Endocan or endothelial cell specific molecule-1 (ESM-1): a potential novel endothelial cell marker and a new target for cancer therapy. Biochim Biophys Acta.

[CR17] Shapiro NI, Trzeciak S, Hollander JE, Birkhahn R, Otero R, Osborn TM, Moretti E, Nguyen HB, Gunnerson KJ, Milzman D, Gaieski DF, Goyal M, Cairns CB, Ngo L, Rivers EP (2009). A prospective, multicenter derivation of a biomarker panel to assess risk of organ dysfunction, shock, and death in emergency department patients with suspected sepsis. Crit Care Med.

[CR18] Skibsted S, Shapiro NI (2014). Transcriptomics may pave the biomarker road in sepsis*. Crit Care Med.

[CR19] Stavrum AK, Petersen K, Jonassen I, Dysvik B (2008). Analysis of Gene-Expression Data Using J-Express. Current Protocols in Bioinformatics/Editoral Board, Andreas D Baxevanis [et al.] Chapter 7:Unit 7 3.

[CR20] Tenna AM, Kappadath S, Stansby G (2012). Diagnostic tests and strategies in venous thromboembolism. Phlebology/Venous Forum of the Royal Society of Medicine.

[CR21] Uitte de Willige S, De Visser MC, Vos HL, Houwing-Duistermaat JJ, Rosendaal FR, Bertina RM (2008). Selectin haplotypes and the risk of venous thrombosis: influence of linkage disequilibrium with the factor V Leiden mutation. J Thromb Haemostasis.

[CR22] Van Bijnen ST, Van Heerde WL, Muus P (2012). Mechanisms and clinical implications of thrombosis in paroxysmal nocturnal hemoglobinuria. J Thromb Haemostasis.

[CR23] Wong HR, Salisbury S, Xiao Q, Cvijanovich NZ, Hall M, Allen GL, Thomas NJ, Freishtat RJ, Anas N, Meyer K, Checchia PA, Lin R, Shanley TP, Bigham MT, Sen A, Nowak J, Quasney M, Henricksen JW, Chopra A, Banschbach S, Beckman E, Harmon K, Lahni P, Lindsell CJ (2012). The pediatric sepsis biomarker risk model. Crit Care.

[CR24] Wong HR, Lindsell CJ, Pettila V, Meyer NJ, Thair SA, Karlsson S, Russell JA, Fjell CD, Boyd JH, Ruokonen E, Shashaty MG, Christie JD, Hart KW, Lahni P, Walley KR (2014). A multibiomarker-based outcome risk stratification model for adult septic shock*. Crit Care Med.

